# Core promoter mutation contributes to abnormal gene expression in bladder cancer

**DOI:** 10.1186/s12885-022-09178-z

**Published:** 2022-01-15

**Authors:** Teng Huang, Jiaheng Li, San Ming Wang

**Affiliations:** Cancer Center and Institute of Translational Medicine, Faculty of Health Sciences, Ministry of Education Frontiers Science Center for Precision Oncology, University of Macau, Taipa, Macau

**Keywords:** Bladder cancer, Core promoter, Gene expression, Mutation

## Abstract

**Background:**

Bladder cancer is one of the most mortal cancers. Bladder cancer has distinct gene expression signature, highlighting altered gene expression plays important roles in bladder cancer etiology. However, the mechanism for how the regulatory disorder causes the altered expression in bladder cancer remains elusive. Core promoter controls transcriptional initiation. We hypothesized that mutation in core promoter abnormality could cause abnormal transcriptional initiation thereby the altered gene expression in bladder cancer.

**Methods:**

In this study, we performed a genome-wide characterization of core promoter mutation in 77 Spanish bladder cancer cases.

**Results:**

We identified 69 recurrent somatic mutations in 61 core promoters of 62 genes and 28 recurrent germline mutations in 20 core promoters of 21 genes, including *TERT*, the only gene known with core promoter mutation in bladder cancer, and many oncogenes and tumor suppressors. From the RNA-seq data from bladder cancer, we observed  altered expression of the core promoter-mutated genes. We further validated the effects of core promoter mutation on gene expression by using luciferase reporter gene assay. We also identified potential drugs targeting the core promoter-mutated genes.

**Conclusions:**

Data from our study highlights that core promoter mutation contributes to bladder cancer development through altering gene expression.

**Supplementary Information:**

The online version contains supplementary material available at 10.1186/s12885-022-09178-z.

## Background

Bladder cancer is the tenth most common cancer worldwide with an estimated 200,000 deaths per year [1]. Incidence rate of bladder cancer is the highest in Europe, especially in Southern European countries including Spain [1]. Urothelial cancer is the most common histologic type of bladder cancer accounting for 90% of all bladder cancers [2]. While environmental contaminants and smoking are known to be the risk factors for bladder cancer [3], knowledge about genetic factor contributing to bladder cancer is limited although altered expression for the genes related to cell cycle, transcription and cytoskeleton was well observed in bladder cancer [4]; mutation altering *TERT* expression was identified in bladder cancer [5]; and differential gene expression was used to classify bladder cancer into sub-groups [6], the mechanisms of the abnormal gene expression in bladder cancer remains largely elusive.

Gene expression is under precise regulation to ensure spatial and temporal expression, in which transcriptional initiation is the gateway [7, 8]. In eukaryotes, transcriptional initiation is controlled by the basal transcriptional machinery composed of cis- and trans-elements in the core promoter region surrounding the transcriptional start site (TSS) [9]. The cis-elements consist of TFIIB recognition element (BRE), TATA box, Initiator element (Inr), downstream promoter element (DPE) etc. and their flanking sequences, and the trans-elements consist of RNA polymerase II, TFIIB and TFIID etc. and co-activators [8]. Mutation in cis sequences can interfere cis-trans interaction, modulate transcriptional initiation and gene expression level, and cause pathogenic consequences [5, 10, 11]. This is best exemplified by the core promoter mutation in *TERT. TERT* codes for telomerase reverse transcriptase involving in telomere structure. Mutation in *TERT* core promoter creates an ETS binding site and causes *TERT* over expression in multiple types of cancer including bladder cancer [5, 11, 12]. Regardless of the importance of core promoter in controlling gene expression, however, *TERT* remains as the only gene with established relationship between core promoter mutation and cancer. The prevalence of cis-mutation in core promoters remains largely unexplored in most cancer types including bladder cancer.

We hypothesized that core promoter mutation contributes to the abnormal gene expression in bladder cancer. Previously, we developed the Exome-based Variant Detection in Core-promoters (EVDC) method [13] for genome-wide core promoter mutation study, and used it in  mapping the core promoter polymorphism in global human populations [14]. In this study, we applied this method to systematically analyze core promoter mutation in bladder cancer by using the exome data from bladder cancer patients. We identified both somatic and germline core promoter mutations in multiple genes and validated their effects on altering gene expression. Our study reveals that core promoter mutation can contribute to the etiology of bladder cancer.

## Methods

### Sources of sequence data

Exome data from Spanish bladder cancer (*n* = 77) and patient-matched blood [15] were from the NCBI Sequence Read Archive (SRA) database (https://www.ncbi.nlm.nih.gov/sra, SRP029936 and SRP029935). Sequences in SRA format were converted into FASTQ format by using NCBI SRA Toolkit utility (version 2.9.1) [16]. Variants called from exome data of the Iberian population in Spain (IBS) (*n* = 107) sequenced by the 1000 Genome Project [17] were used as the normal population control in the study. Human genome reference sequences were used as the references for core promoter mapping analysis [18, 19] (hg19, https://hgdownload.soe.ucsc.edu/downloads.html#human).

### Identification of core promoter mutations

Core promoter sequences were collected from the exome sequences by using the EVDC method [13]. Core promoter coordinates and sequences from hg19 were extracted by using BEDTools utility (version 2.27.1) [20]. BWA utility (version 0.7.17) was used to map exome sequences to hg19 [21]. The resulting SAM files were converted into BAM files and sorted by using SAMtools utility (version 1.9) [22, 23]. Duplicates were removed by using Picard tools (version 2.18.25), and the read group information was added [24]. The BAM files were further processed by using GATK (version 4.1.1.0) [24] with its recommended best practices pipeline. The called mutation files were compressed and indexed by using BCFtools utility (version 1.9) [22, 23], and annotated by using ANNOVAR [25]. Normal polymorphism in cancer samples were removed by filtering the normal population variation data including the Iberian population in Spain (IBS) sequenced by the 1000 Genome Project. Mutations with MAF value > 0.01 were also eliminated [26, 27]. Mutations absent in annotation data sets (dbSNP, 1000 Genome, ESP6500, ExAC, gnomAD, COSMIC, ClinVar) were classified as novel variants. The variants present in at least two cases were regarded as recurrent variants and used for further analysis. Somatic and germline mutations were distinguished by comparing the mutations from the tumor and the paired blood samples [15]. Examples of sequencing chromatograms were displayed by Tablet software [28].

### Gene expression in human tissues

RNA-seq data of bladder cancer and adjacent normal tissues generated by TCGA [29] were from the cBioPortal database [30] for differential gene expression analysis (https://cbioportal-datahub.s3.amazonaws.com/blca_tcga_pan_can_atlas_2018.tar.gz). Differentially expressed genes were identified by using Student’s t-test and fold changes. Gene identifiers were converted by using SynGO [31]. Volcano plots showing differential expressed genes were generated by using R ggplot2 package [32]. The expression for the luciferase reporter assay-tested genes in human tissues were searched in Human Protein Atlas [33].

### Luciferase reporter assay

Human embryonic kidney 293 cells (HEK 293) were used to test the effects of core promoter mutation in gene expression using the dual-luciferase reporter system. Cells were grown in Dulbecco’s modified Eagle’s media/Nutrient Mixture culture medium with 10% fetal bovine serum, 100 IU/ml penicillin and 100 IU/ml streptomycin sulfate. The wild-type and mutated core promoter sequences were synthesized, cloned into pGL3 luciferase reporter vector, and validated by Sanger sequencing (BGI TECH SOLUTIONS, Beijing, China). Fifty nicrogram of pGL3 containing the targeted core promoter sequences and 5 μg of control pRL *Renilla* luciferase reporter vector were mixed, and co-transfected into HEK 293 cells by using Lipofectamine 3000 Transfection Reagent (Thermo Fisher SCIENTIFIC, MS, USA). Forty-eight hours after the transfection, cells were harvested to measure luciferase activity by using the Dual-Luciferase Reporter Assay System (Promega, WI, USA) following the instruction (PerkinElmer Victor X3 Microplate Reader, OH, USA). Three independent tests were performed for each core promoter. Luciferase activity was normalized by dividing *firefly* luciferase activity with *Renilla* luciferase activity:

$$E_l=E_f/E_r$$

*E*_*f*_: *firefly* luciferase activity, *E*_*r*_: *Renilla* luciferase activity, *E*_*l*_: normalized luciferase activity.

### Characterization of core promoter mutation-affected genes

For the core promoter mutated genes, their function categories and involved pathways were analyzed by using GO (Gene Ontology) knowledgebase [34] and GeneCards database [35]. Candidate drugs targeting the core promoter mutated genes were identified in DrugBank [36]. GO terms and drugs were identified by using Metascape [37]. Expression Quantitative Trait Loci in PancanQTL database [38] was used to test the effects of the core promoter-mutated genes on gene expression in bladder tissue. A cancer driver gene panel was generated by integrating the 1064 cancer driver genes in OncoKB [39] database and the 299 genes from previous cancer driver gene study [40], and the core promoter mutated genes were searched in this gene panel to identify potential driver genes with core promoter mutation. KEGG (Kyoto Encyclopedia of Genes and Genomes) database [41] was used to identify the pathways affected by the mutated driver genes.

### Statistics analysis

In the analysis of differential gene expression and dual-luciferase reporter assay, *p*-value < 0.05 by using Student’s t-test, and fold changes ≥1.5 were considered as significantly different. Student’s t-test were calculated by using T.TEST function in MS EXCEL. In the enrichment analysis, p-value < 0.05 by using the accumulative hypergeometric test, overlap ≥1 and enrichment factor > 1.5 were considered as significantly different. Statistics test in enrichment analysis was calculated by Metascape.

## Results

### Core promoter mutation in bladder cancer

**Fig. 1:**
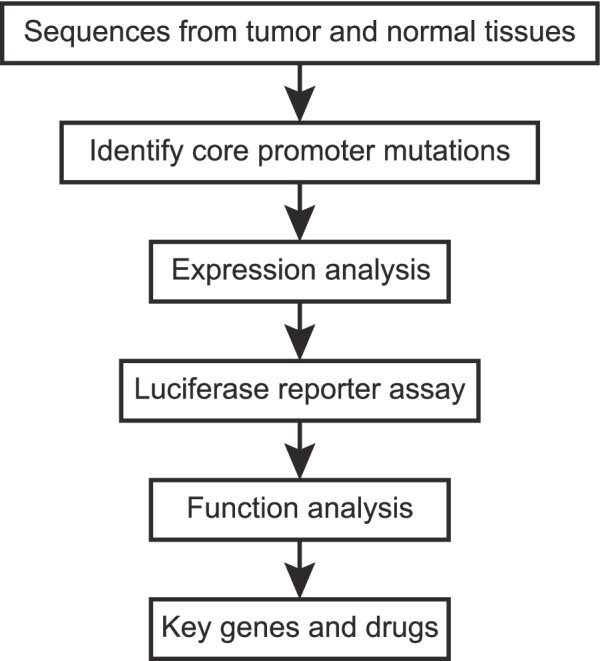
Scheme of the analytic process

We collected the core promoter sequences from a total of 77 tumor samples and matched blood samples from the exome data generated by the Spanish urothelial bladder cancer study [15]. We called variants from the collected core promoter sequences (Fig. S1), removed polymorphic variants through filtering the variants from normal human population including the IBS population, and identified somatic and germline mutations by comparing the variants between cancer and blood samples. Figure 1 outlines the analytic process of the  study.

We identified a total of 216 recurrent somatic mutations (present in ≥2 carriers), 3 mutations per cancer case on average, composed of 69 distinct mutations in 61 core promoters of 62 genes (Table 1A, Table S1A and Table S2A, B). Of the 69 somatic mutations, 45 (65.2%) were substitution, 14 (20.3%) were deletion and 10 (14.5%) were insertion (Table 1B); 63 (91.3%) were absent in the COSMIC database and 37 (53.6%) were novel and absent in all mutation databases; and 8 (11.6%) were located at simple repetitive sequences.

**Table 1:** Summary of core promoter mutations identified in bladder cancer

Items	Core promoter variants	
	Somatic	Germline
A. General features		
Total	216	88
Average number of mutation/case	3	1
Distinct	69	28
Co-promoter with variants	61	20
Gene affected	62	21
Absent in COSMIC database	63	28
Novel	37	15
Non-repetitive	61	19
Repetitive	8	9
B. Type		
Total	69	28
Substitution	45	18
Insertion	10	3
Deletion	14	7
C. Mutation located in core promoter motifs		
Total^a^	86	21
MTE_box2	23	3
DPE	10	4
Inr	9	2
Ets	9	–
DTIE	6	1
TCT	4	1
BREu	3	–
TATA box	2	1

^a^Some mutations affected > 1 motif

We also identified a total of 88 recurrent germline mutations, 1 mutation per cancer case on average, composed of 28 distinct mutations in 20 core promoters of 21 genes (Table 1A, Table S1B and Table S2C, D). Of the 28 germline mutations, 18 (64.3%) were substitution, 7 (25%) were deletion and 3 (10.7%) were insertion (Table 1B); 15 (53.6%) were novel; and 9 (32.1%) were located at simple repetitive sequences.

We observed that the core promoter mutations were enriched in multiple core promoter motifs (Table 1C and Table S3). For example, MTE box2 motif had 23 somatic mutations and 3 germline mutations. Reflecting the fact that TATA box is not tolerable for base changes [13], only 2 somatic and 1 germline mutations were located at the TATA box. This also served as an internal control in validating the reliability of the mutations identified in the bladder cancer from this study.

### Effects of core promoter variation on gene expression

**Fig. 2:**
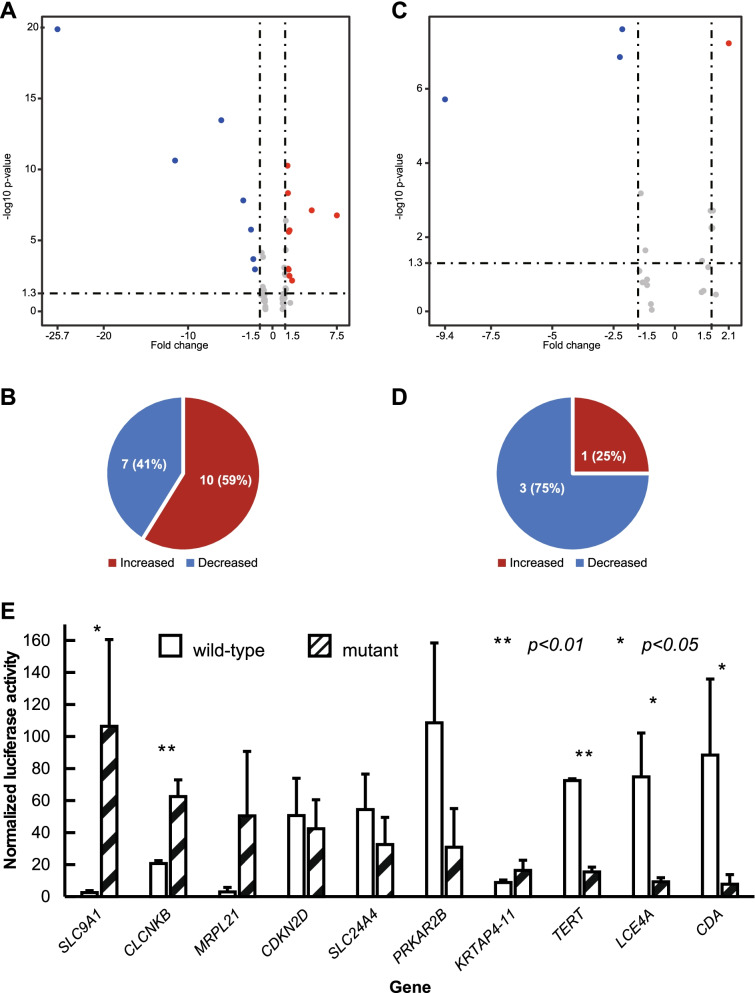
Core promoter mutated genes with altered gene expression in bladder cancer. The volcano plots showed the altered expression of core promoter mutated genes between cancer and adjacent normal samples based on RNA-seq data. X-axis represented fold changes of increased or decreased expression, and Y-axis represented distribution of the genes with altered expression at -log10 scale. The pie charts displayed the number of gene with altered expression. **A.** altered expression of somatic core promoter mutated genes; **B.** somatic core promoter mutated genes with altered expression; **C.** altered expression of germline core promoter mutated genes; **D.** germline core promoter mutated genes with altered expression. **E.** luciferase activities with mutated core promoters. Luciferase activities in 10 mutated core promoters were compared with the corresponding wild-type core promoters. Three independent tests were performed for each core promoter. *refers to these with significant differences

To address if core promoter mutation could lead to altered expression of the core promoter-mutated genes, we compared the RNA-seq data between bladder cancer and adjacent normal samples. Of the core promoter somatically mutated 62 genes, 17 (27.4%) were significantly different including 10 increased and 7 decreased expressions. Of the 17 genes, *TERT* had the highest of 7.5-fold increased expression and *CFD* had the highest of 25.7-fold decreased expression. Of the core promoter germline-mutated 21 genes, 4 (19.0%) were significantly different including 1 increased and 3 decreased expressions (Fig. 2A-D and Table S4). We also searched the Human Protein Atlas database to collect the expression information for the core promoter mutation-affected genes in normal and bladder cancer (Table S5A). The result showed that *TERT* was not expressed in normal bladder but overexpressed in bladder cancer with core promoter C228T mutation *TERT* [5]; survival data of *CDA*, *SLC9A1* and *SLC24A4* also showed that their expression levels were associated with 5-year survival significantly.

While the data from the RNAseq data analysis provided evidence for the impact of the core promoter mutation on expression, the information was indirect as the genes in the original samples could not be sure to contain the core promoter mutations except *TERT*. Therefore, we used reporter gene assay to test the effects of core promoter mutation in gene expression. Based on the considerations 1) the functional importance of the genes carrying the mutation, 2) significance of the altered expression level by expression data analysis, and 3) core promoter sequence features for designing and constructing the mutants, we selected 10 core promoters for the test, including *TERT*, *CDA*, *SLC9A1*, *SLC24A4*, *PRKAR2B*, *CDKN2D*, *CLCNKB*, *LCE4A*, *KRTAP4–11* and *MRPL21*. The canonical core promoter mutation in *TERT* was selected as internal standard. *CDA* involves in metabolic process, *SLC9A1* is related with cancer growth, *SLC24A4* had decreased expression in bladder cancer. *PRKAR2B* is involved in mitotic cell cycle transition and response to cancer-related drug clozapine. *CDKN2D* is involved in cell cycle, metabolic process, and nutrient response. *CLCNKB* regulates trans-membrane transport and trans-differentiation. *LCE4A and KRTAP4–11* are related with cellular differentiation. *MRPL21* is related to mitochondrion metabolism. Each mutated core promoter was paired with the corresponding wildtype core promoter control for the test. We generated the mutated core promoters for the 10 selected genes, cloned into luciferase reporter constructs. Each type of mutant construct was transfected into 293 cells, the luciferase activities were compared with the corresponding wild-type core promoter controls. Of the 10 mutated core promoters tested, 5 had significantly altered luciferase activities (*SLC9A1*, *CLCNKB*, *TERT*, *LCE4A* and *CDA*, *p*-value < 0.05), of which *SLC9A1* and *CLCNKB* had increased luciferase activities, *TERT*, *LCE4A* and *CDA* had decreased luciferase activities (Fig. 2E and Table S5B).

### Cancer driver genes and pathways affected by core promoter mutation

**Fig. 3:**
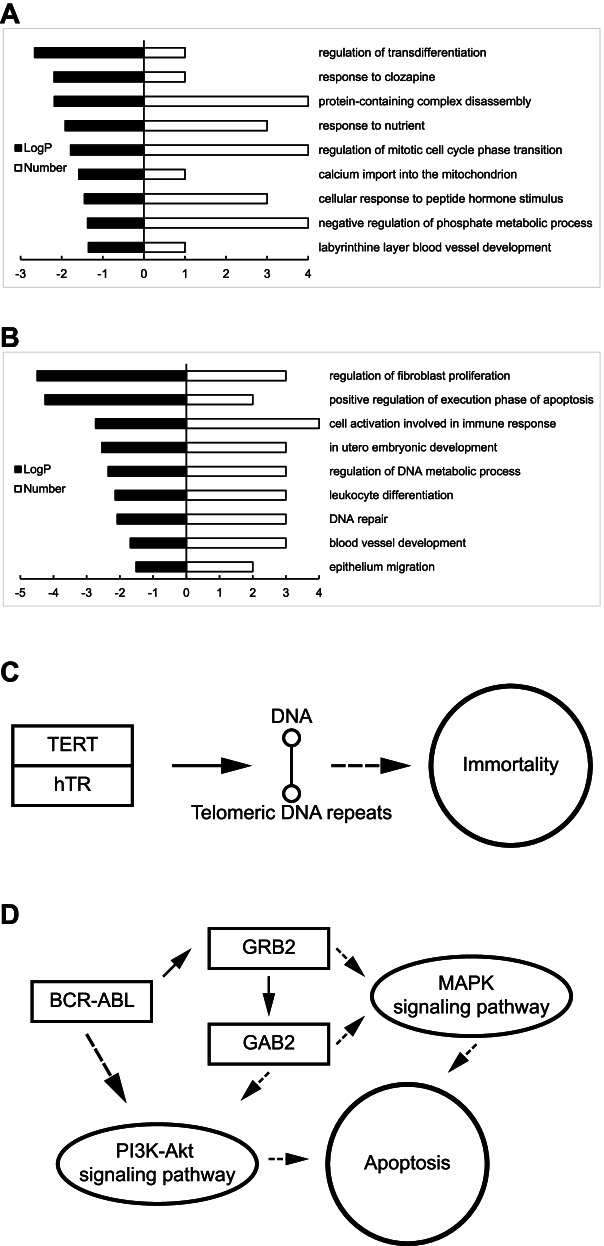
GO classification and KEGG pathways of core promoter-mutated genes. **A.** GO classification of somatic core promoter-mutated genes; **B.** GO classification of germline core promoter-mutated genes; **C.** KEGG pathway of *TERT* involved in cancer (https://www.kegg.jp/pathway/map05200). The C228T in the core promoter of *TERT* generated a new Ets binding motif, altered *TERT* expression, and promoted cellular immortality. **D.** KEGG pathway of *GAB2* involved in cancer (https://www.kegg.jp/pathway/ko05220). GAB2 involves in MAPK and PI3K-Akt signal pathways in immune-response and apoptosis. A germline A > C mutation at − 60 altered the sequence from “CCCACC” to “CCCCCC”, caused decreased GAB2 expression in bladder cancer (Table S7). Black bar: statistical significance of gene group; white bar: number of genes enriched in the group; full arrow: direct effects; dotted line arrow: indirect effects

By Gene Ontology analysis, we observed that the core promoter-mutated genes were enriched in the functional pathways highly relevant to oncogenesis (Fig. 3 and Table 2). For example, somatic mutated genes were enriched in “Regulation of mitotic cell cycle phase transition”, “Cellular response to peptide hormone stimulus” and “Selective advantage”; germline mutated genes were enriched in “Evading apoptosis”, “Evading the immune system”, “Tissue invasion and metastasis” and “DNA repair”; and both somatic and germline-mutated genes were enriched in “Deregulated metabolism”, “Differentiation” and “Sustained angiogenesis” (Table S6).

**Table 2:** Examples of functional important genes with core promoter mutation

Items	Mutation	Co-promoter position	#Carrier	Expression
A. Pathways with core promoter mutated genes				
Deregulated metabolism	Somatic Germline			
Differentiation	Somatic Germline			
Sustained angiogenesis	Somatic Germline			
Regulation of mitotic cell cycle phase transition	Somatic			
Cellular response to peptide hormone stimulus	Somatic			
Selective advantage	Somatic			
Evading apoptosis	Germline			
Evading the immune system	Germline			
Tissue invasion and metastasis	Germline			
DNA repair	Germline			
B. Examples of cancer related genes				
*TERT*	Somatic	−66	3	+ 7.5
*PRKAR2B*	Somatic	93	2	−6.2
*SMUG1*	Germline	90	2	+ 2.1
*GAB2*	Germline	−60	3	−2.3

We compared the core promoter-mutated genes with altered gene expression to the cancer driver gene list and observed that the somatic-mutated *TERT* and *PRRX1*, and germline-mutated *GAB2* were on the list (Table S7). *TERT* is the only known gene with somatic core promoter mutation in bladder cancer [5]. TERT participates in the formation of telomeric DNA repeats and affects the immortality of cell (Fig. 3C). The C228T in the core promoter of *TERT* was detected in 3 bladder cancer cases but absent in all paired blood samples, and no coding mutation in *TERT* was detected. The mutation generated a new binding motif of the Ets transcription factor, deleted a DTIE and created a new putative DCE_box1, and caused 4.8-fold decreased expression as shown by luciferase report gene assay (Fig. 2E and Table S5B). PRRX1 is a transcription co-activator enhancing DNA-binding activity of SRF (serum response factor) required for the induction of multiple genes by growth and differentiation factors. A CT-track simple repetitive sequence was inserted into the CT-repeat region in the core promoter, caused decreased *PRRX1* expression in bladder cancer. GAB2 involves in immune-response and apoptosis (Fig. 3D). A germline A > C mutation at − 60 altered the sequence from “CCCACC” to “CCCCCC”, caused decreased expression in bladder cancer as shown by RNA-seq data (Table S7).

### Potential drugs targeting core promoter mutated genes

The core promoter mutation-affected genes with altered expression provide potential drug targets for bladder cancer treatment [42]. From the DrugBank, we identified 6 drugs/compounds targeting 3 somatic-mutated genes with altered expression (1 increased and 2 decreased expression) (Table S8). For example, an approved drug Zidovudine targets *TERT* by inhibiting telomerase activity [43]; cyclic adenosine monophosphate (cAMP) targets *PRKAR2B*, which is a regulatory subunit of the cAMP-dependent protein kinases. We also identified 10 drugs/compounds targeting germline-mutated PDE10A (LINC00473) with decreased expression (Table S8), including Dipyridamole acting as a phosphodiesterase inhibitor to suppress PDE10A activity [44] and Triflusal, an antagonist to PDE10A [45].

## Discussion

Core promoter variation is well recognized in affecting gene expression. However, the role of core promoter mutation in oncogenesis has not been well established. With its distinct gene expression in bladder cancer, our study identified both somatic and germline mutations in the core promoters of a group of cancer-related genes. Our study highlights that core promoter mutation can be an important etiological factor in bladder cancer oncogenesis through altering the expression of cancer genes.

In our study, the somatic and germline mutations were identified by 1) Comparing the cancer samples with their paired blood samples from the same study; 2) Filtering the called variant data by variants from normal human populations including these from the local population to eliminating normal polymorphism; 3) Using the exome data and RNA-seq data from urothelial cancer for mutation and expression analysis; 4) Comparing altered expression between cancer and adjacent normal samples. These steps ensured high reliability of the mutations identified by our study, as examplified by the identification of core promoter mutation in *TERT*, which is known to be present in bladder cancer [11]. It is interesting to note that the core promoter-mutated *TERT* causes its increased expression in multiple types of cancer [5, 11], including in our expression analysis (Table S4). However, in core promoter mutated *TERT*-luciferase reporter assay, the mutation caused decreased luciferase expression (Table S5B). This could be related to the differences of cell types, in vitro and in vivo conditions, etc., which may haved different regulation mechanisms of transcription initiation [46]. As a widely reported oncogene with core promoter mutation, the opposite effects of the mutated *TERT* core promoter on gene expression is worth of further study. In *TP53* core promoter, we found a germline mutation C > T at + 101 and a poly T track deletion at + 95, but no expression change was observed between cancer and control as shown by RNA-seq data analysis.

Our study identified multiple novel core promoter mutated genes. For example, somatic mutations were identified in the core promoter of *PRKAR2B*, and germline mutations were identified in the core promoter of *SMUG1* and *GAB2*. Gene ontological and pathway analysis showed that these core promoter mutated genes are oncogenic through affecting multiple functional pathways: SMUG1 participates in DNA repair (KEGG: hsa03410); GAB2 contributes to cellular differentiation, immunity and cancer (KEGG: ko05220); PRKAR2B regulates mitotic cell cycle transition and metabolism (KEGG: hsa04910). Simple repetitive sequence is widely present in promoter, and plays important role in gene expression regulation [47]. The core promoter mutation in *GAB2* and *PRRX1* occurred at simple repetitive sequences, caused their altered expression in cancer. It is interesting to notice that both somatically mutated *PRKAR2B* and germline-mutated *GAB2* were present in a single bladder cancer case (BioSample accession number: SAMN02351138). Somatic mutation in *PRKAR2B* created putative motifs in the core promoter, caused *PRKAR2B* differentially expressed, affected regulation of mitotic cell cycle transition and phosphate metabolism [48]. *GAB2* is a cancer driver gene. The high frequent germline mutation in *GAB2* was also present in acute myeloid leukemia in the International Cancer Genome Consortium study and in acute lymphoblastic leukemia with *Ras*-independent leukemogenic effects [49]. Drug targeting the core promoter-mutated gene offers a potential pharmacological theraputic agent for bladder cancer treatment and worthy to be studied further.

## Conclusions

Our study identified both somatic and germline mutations in core promoters of multiple cancer driver genes in bladder cancer, highlighting that altered regulatory machinery including the core promoter can contribute to the alterative gene expression in cancer.

## Supplementary Information

**Additional file 1: Fig S1.** Sequence chromatograms of three core promoter mutations. A. Mutation T > C/TC > CA (chr1:152,681,543-152,681,544) in core promoter of *LCE4A* occurred in 79 out of 80 reads in a sample. B. Mutation A > G/AG > GA (chr1:20,915,531-20,915,532) in core promoter of *CDA* occurred in 28 out of 68 reads in a sample. C. Mutation C > G (chr11:75,110,552-75,110,552) in core promoter of *RPS3* occurred in 17 out of 77 reads in a sample. Top line: reference sequences; other lines: sequence reads mapped to the reference sequences; base marked in red: the base different from the reference sequences; arrow: the mutated base identified by sequence alignment.

**Additional file 2: Table S1.** A. List of somatic non-repetitive core promoter mutations. B. List of germline non-repetitive core promoter mutations.

**Additional file 3: Table S2.** A. List of somatic core promoter mutations in simple repetitive sequences. B. Type of somatic core promoter mutations in simple repetitive sequences. C. List of germline core promoter mutations in simple repetitive sequences. D. Type of germline core promoter mutations in simple repetitive sequences.

**Additional file 4: Table S3.** Frequency of core promoter mutations in motifs.

**Additional file 5: Table S4.** Core promoter mutated genes with altered gene expression.

**Additional file 6: Table S5.** A. Expression for the luciferase reporter assay-tested genes in human tissues. B. Expression of core promoter mutated genes in luciferase reporter assay.

**Additional file 7: Table S6.** A. GO classification of somatic core promoter-mutated genes. B. GO classification of germline core promoter-mutated genes.

**Additional file 8: Table S7.** Driver gene with core promoter mutation.

**Additional file 9: Table S8.** Drugs targeting core promoter-mutated genes.

## Data Availability

All data generated or analyzed during this study are included in this published article and its supplementary information files.

## Abbreviations

BRE: TFIIB recognition element
DPE: Downstream promoter element
EVDC: Exome-based Variant Detection in Core-promoters
FPKM: Number Fragments Per Kilobase of exon per Million reads
GO: Gene Ontology
HEK 293: Human embryonic kidney 293 cells
IBS: Iberian population in Spain
Inr: Initiator element
KEGG: Kyoto Encyclopedia of Genes and Genomes
pTPM: Transcripts per million protein coding genes
SRA: Sequence Read Archive
SRF: Serum response factor
TSS: Transcriptional start site
