# Supplementary figures and images for: Core promoter mutation contributes to abnormal gene expression in bladder cancer
Source: BMC Cancer. 2022 Jan 15;22:68. doi: 10.1186/s12885-022-09178-z (PMC8761283; doi:10.1186/s12885-022-09178-z)

Fig. S1

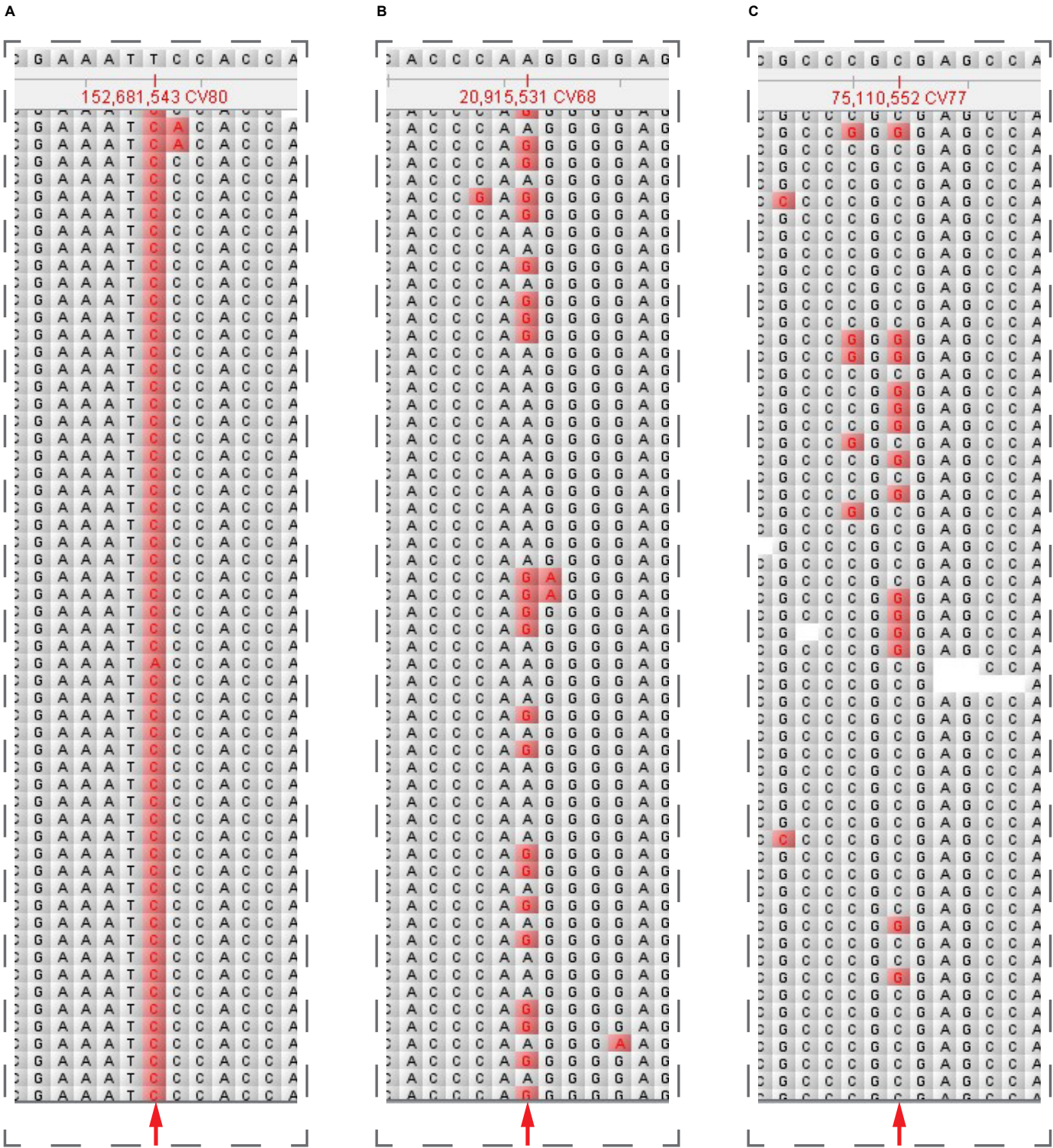

Supplement: Supplementary file 1 — Additional file 1: Fig S1. Sequence chromatograms of three core promoter mutations. A. Mutation T > C/TC > CA (chr1:152,681,543-152,681,544) in core promoter of LCE4A occurred in 79 out of 80 reads in a sample. B. Mutation A > G/AG > GA (chr1:20,915,531-20,915,532) in core promoter of CDA occurred in 28 out of 68 reads in a sample. C. Mutation C > G (chr11:75,110,552-75,110,552) in core promoter of RPS3 occurred in 17 out of 77 reads in a sample. Top line: reference sequences; other lines: sequence reads mapped to the reference sequences; base marked in red: the base different from the reference sequences; arrow: the mutated base identified by sequence alignment. [file 12885_2022_9178_MOESM1_ESM.pdf]
